# Multi-species coexistence in Lotka-Volterra competitive systems with crowding effects

**DOI:** 10.1038/s41598-017-19044-9

**Published:** 2018-01-19

**Authors:** Maica Krizna A. Gavina, Takeru Tahara, Kei-ichi Tainaka, Hiromu Ito, Satoru Morita, Genki Ichinose, Takuya Okabe, Tatsuya Togashi, Takashi Nagatani, Jin Yoshimura

**Affiliations:** 1 Graduate School of Science and Technology, Shizuoka University, 3-5-1 Johoku, Naka-ku Hamamatsu, 432-8561 Japan; 2 Mathematics Division, Institute of Mathematical Sciences and Physics, University of the Philippines Los Baños, College, Laguna, 4031 Philippines; 3 Graduate School of Integrated Science and Technology, Shizuoka University, 3-5-1 Johoku, Naka-k Hamamatsu, 432-8561 Japan; 4 Department of Mathematical and Systems Engineering, Shizuoka University, 3-5-1 Johoku, Naka-k Hamamatsu, 432-8561 Japan; 5 Department of Genaral Systems Studies, University of Tokyo, 3-8-1 Komaba, Meguro Tokyo, 153-8902 Japan; 6 Department of International Health, Institute of Tropical Medicine, Nagasaki University, Nagasaki, 852-8523 Japan; 7 Marine Biosystems Research Center, Chiba University, 1 Uchiura, Kamogawa, Chiba, 299-5502 Japan; 8 Department of Mechanical Engineering, Shizuoka University, 3-5-1 Johoku, Naka-ku Hamamatsu, 432-8561 Japan; 9 Department of Environmental and Forest Biology, State University of New York College of Environmental Science and Forestry, Syracuse, NY 13210 USA

## Abstract

Classical Lotka-Volterra (LV) competition equation has shown that coexistence of competitive species is only possible when intraspecific competition is stronger than interspecific competition, i.e., the species inhibit their own growth more than the growth of the other species. Note that density effect is assumed to be linear in a classical LV equation. In contrast, in wild populations we can observed that mortality rate often increases when population density is very high, known as crowding effects. Under this perspective, the aggregation models of competitive species have been developed, adding the additional reduction in growth rates at high population densities. This study shows that the coexistence of a few species is promoted. However, an unsolved question is the coexistence of many competitive species often observed in natural communities. Here, we build an LV competition equation with a nonlinear crowding effect. Our results show that under a weak crowding effect, stable coexistence of many species becomes plausible, unlike the previous aggregation model. An analysis indicates that increased mortality rate under high density works as elevated intraspecific competition leading to the coexistence. This may be another mechanism for the coexistence of many competitive species leading high species diversity in nature.

## Introduction

Competition is one of the fundamental ecological interactions between species^1^. We can observe that coexisting species are competing for the same resources^2^. A typical resource competition model which has been recognized widely is the Classical Lotka-Volterra competition^1,2^. The analyses of this equation show that coexistence of two or more species becomes only possible if intraspecific competition is stronger than interspecific competition^3^. Otherwise, dynamics leads to the exclusion of one species among *n* species, known as the competitive exclusion principle^1,4^. However, in natural communities many competing species have been coexisting in the same habitat over time, resulting in a high species diversity. Hence, we suspect that there should be some mechanisms for coexistence of competitive species, e.g., spatial structures^5,6^. These models, however, introduce an additional complexity into the mathematical models of classical LV systems. Compared with these complex models, coexistence of multiple species in natural communities seems to be far more ubiquitous. Therefore, a more universal explanation may be worth considering.

Crowding effect is considered as one of the ubiquitous mechanisms in any biological populations^7–16^. A nonlinear density effect at high densities is called crowding effect, while that at low densities, Allee effect^7–17^. Unlike this nonlinear density effect, in the traditional mathematical models of population dynamics, density effect is usually treated as constant (i.e., linear), and crowding effect is not included. To consider the nonlinear density effects, aggregation models have been developed and studied extensively introducing ‘mean crowding’^8–10,15,16^. These models show the coexistence of a few species, but not many species. The ‘mean crowding’ is a statistical feature of crowding affecting population growth rate (both birth rate and mortality). Here, we develop a simple LV type competition model with crowding effect on mortality only. We assume that the mortality rate of an individual increases with the density of a population. Moreover, using our modified LV competition model with nonlinear crowding effect, we show that multiple species are generally possible to coexist using LV system with crowding effect. This coexistence dynamics should be applicable to insect or animal species.

## Results

**Figure 1:**
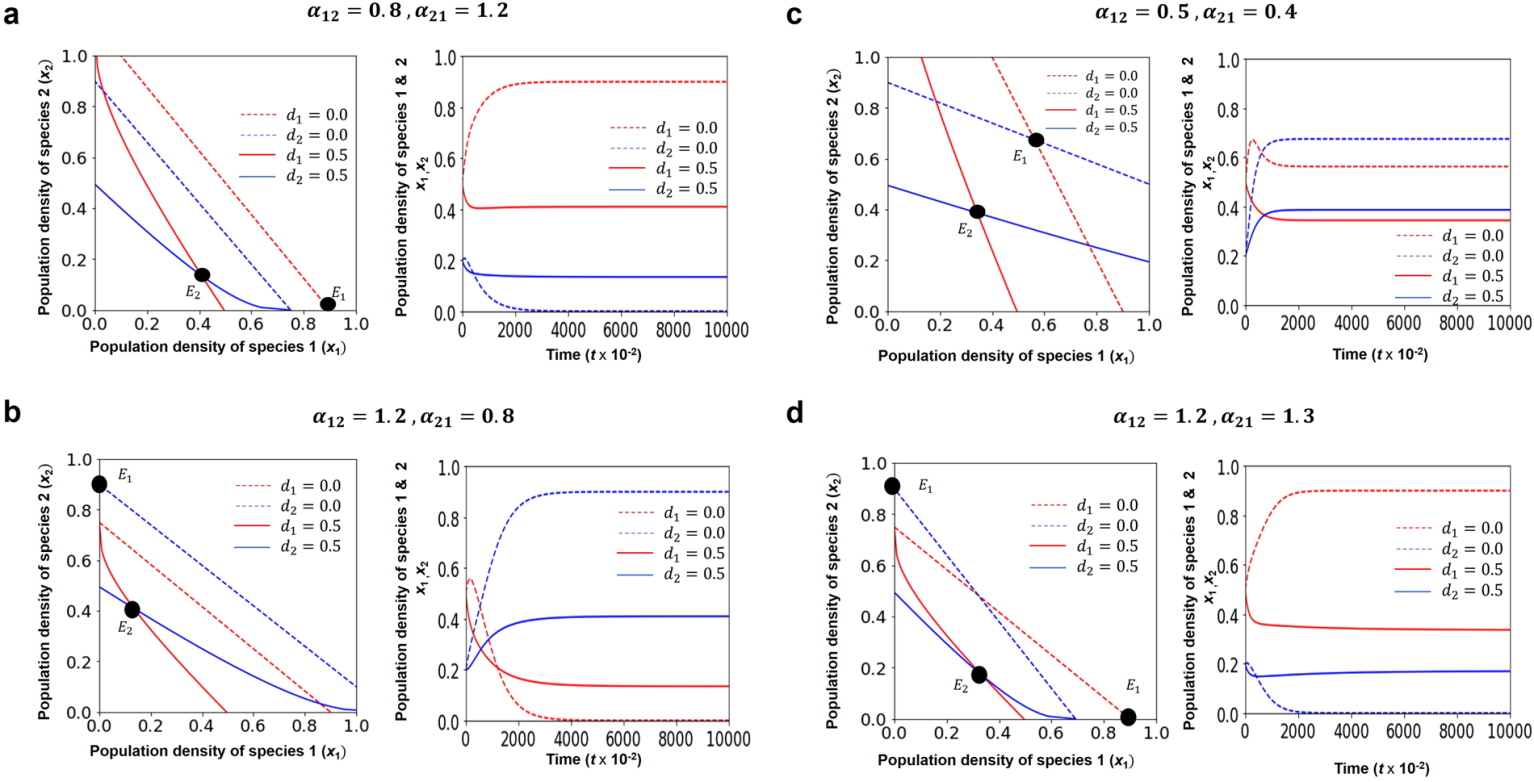
The zero isoclines and the long-term variation density of the classical LV competition equation with the inclusion of crowding effect for the four classical cases. Crowding effects (Solid line: *d*_*i*_ = 0.5, equilibrium: *E*_2_) enable coexistence in all four cases of classical LV system (Broken line: *d*_*i*_ = 0.0, equilibrium: *E*_1_). (**a**) $({K}_{2} < \frac{{K}_{1}}{{\alpha }_{12}},{K}_{1} > \frac{{K}_{2}}{{\alpha }_{21}})$, (**b**) $({K}_{2} > \frac{{K}_{1}}{{\alpha }_{12}},{K}_{1} < \frac{{K}_{2}}{{\alpha }_{21}})$, (**c**) $({K}_{2} < \frac{{K}_{1}}{{\alpha }_{12}},{K}_{1} < \frac{{K}_{2}}{{\alpha }_{21}})$, and (**d**) $({K}_{2} > \frac{{K}_{1}}{{\alpha }_{12}},{K}_{1} > \frac{{K}_{2}}{{\alpha }_{21}})$. Colors indicate *x*_1_(red) and *x*_2_ (blue). *K*_*i*_: carrying capacity of species *i*, and *α*_*ij*_: competition coefficient from species *j* to species *i*. Parameters value: *b*_*i*_ = 1.0, *d*_*i*_ = 0.5, *m*_*i*0_ = 0.1, 0 ≤ *δ* ≤ 5. (**a**) *α*_12_ = 0.8, *α*_21_ = 1.2. (**b**) *α*_12_ = 1.2, *α*_21_ = 0.8. (**c**) *α*_12_ = 0.5, *α*_21_ = 0.4. (**d**) *α*_12_ = 1.2, *α*_21_ = 1.3. Initial density *x*_1_ = 0.5, *x*_2_ = 0.2. We used Anaconda package of the software Python 3.6 for our simulation analysis^22^.

We consider the LV competition system where all interspecific competition rates are unity, i.e., *α*_*ij*_ = *α*_*ji*_ = 1∀*i*. Setting *dx*_*i*_/*dt* = 0, we obtain the zero isoclines for the modified Lotka-Volterra (LV) competition equations with nonlinear crowding effect rate *m*_*i*_. These isoclines are straight lines in the classical LV competition equation when we sketch the graph of the population density 1 with respect to the population density 2 (Fig. 1, dotted lines). However, we observed that these isoclines turn out to be curved when we include a nonlinear crowding effect *m*_*i*_ (Fig. 1, solid lines). All four cases of Lotka-Volterra model show convergent-stable coexistence by adding a crowding effect, where an inferior (superior) species always increases (decreases) in densities (Fig. 1). The equilibrium point is moving from *E*_1_(*d*_*i*_ = 0.0) to *E*_2_(*d*_*i*_ = 0.5) which is also caused by the inclusion of a crowding effect *m*_*i*_, irrespective of other constant parameters (Fig. 1).

**Figure 2:**
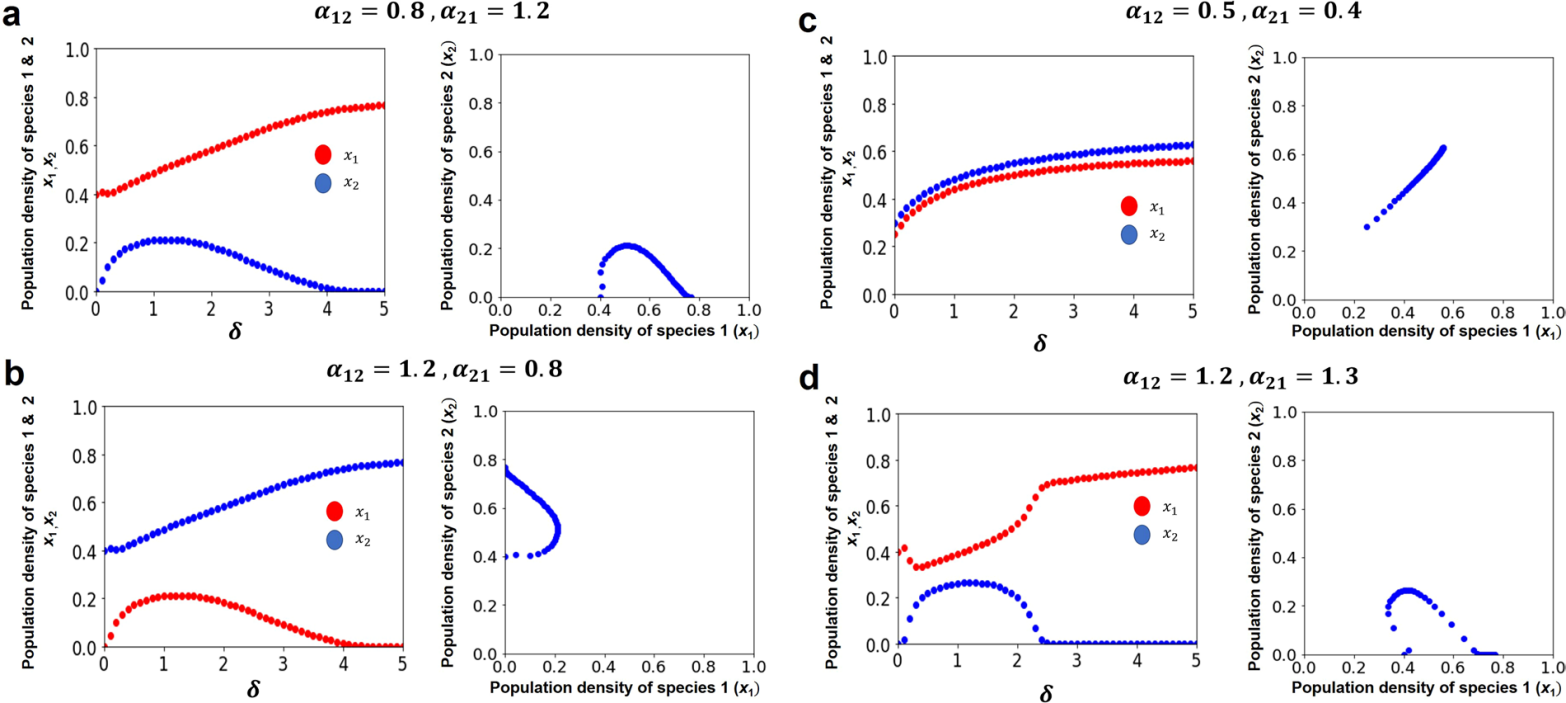
Modified LV competition system with nonlinear crowding effect using the four possible cases of isoclines for the crowding strength factor *δ*. All non-coexistence patterns (**a,b,d**) move to coexistence as *δ* → 1.0 and return to competitive exclusion as *δ* → 5.0. The right column figures show the equilibrium points in the *x*_1_ − *x*_2_ phase plane. (**a**) $({K}_{2} < \frac{{K}_{1}}{{\alpha }_{12}},{K}_{1} > \frac{{K}_{2}}{{\alpha }_{21}})$, (**b**) $({K}_{2} > \frac{{K}_{1}}{{\alpha }_{12}},{K}_{1} < \frac{{K}_{2}}{{\alpha }_{21}})$, (**c**) $({K}_{2} < \frac{{K}_{1}}{{\alpha }_{12}},{K}_{1} < \frac{{K}_{2}}{{\alpha }_{21}})$, and (**d**) $({K}_{2} > \frac{{K}_{1}}{{\alpha }_{12}},{K}_{1} > \frac{{K}_{2}}{{\alpha }_{21}})$. Colors indicate *x*_1_(red) and *x*_2_ (blue). Parameter value and software used: see Fig. 1.

**Figure 3:**
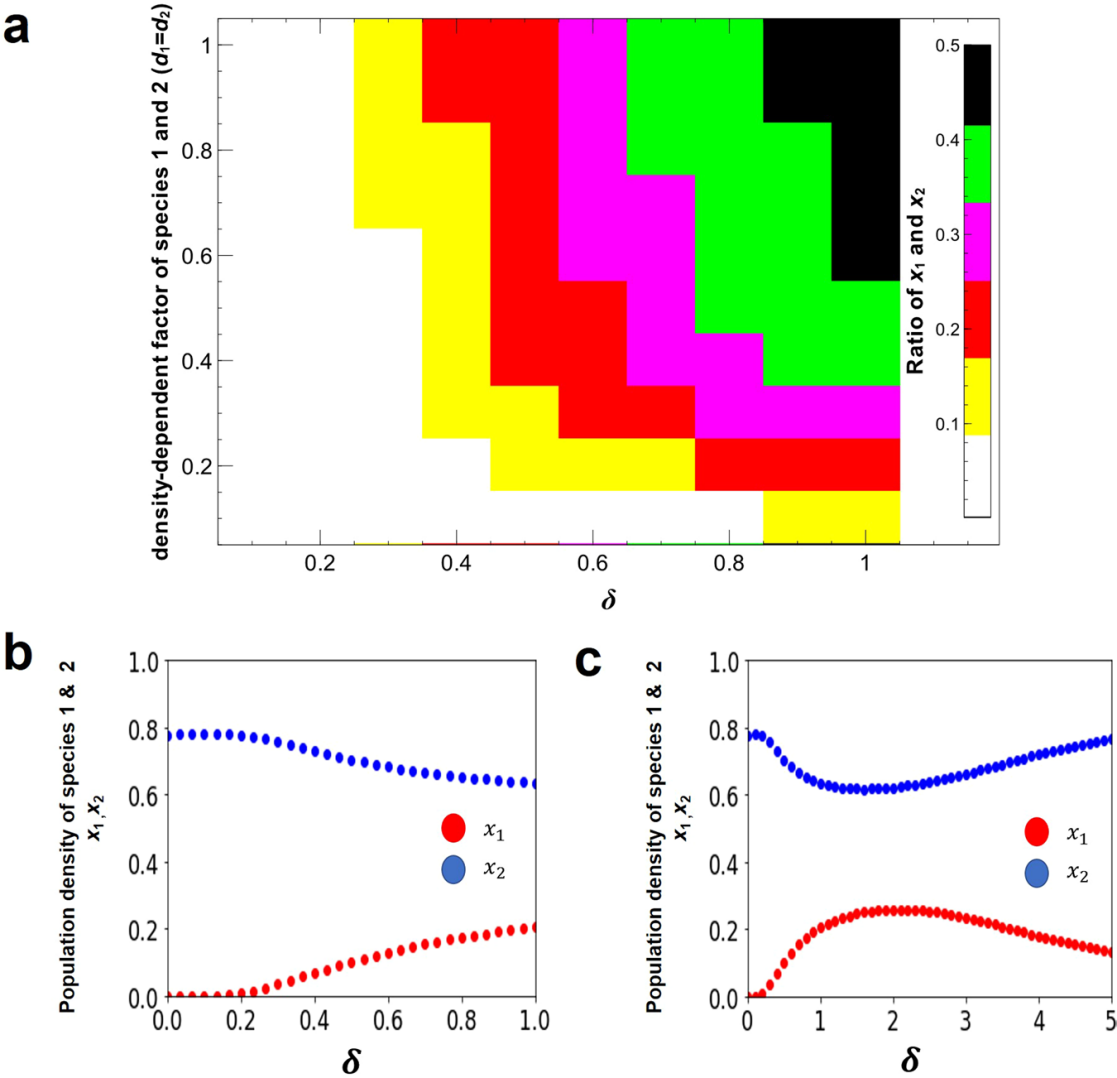
The effects of *δ* on population density in LV competition model with crowding effect. (**a**) Density ratio (*x*_1_/*x*_2_) of species 1 and 2 plotted on the crowding strength factor *δ* and the basic crowding component constant assuming equal between species 1 and 2 (*d*_1_ = *d*_2_). (**b**,**c**) Density of two species (*x*_1_: red, *x*_2_: blue) plotted against *δ*, where (**b**) 0 ≤ *δ* ≤ 1, (**c**) 0 ≤ *δ* ≤ 5, and *d*_*i*_ = 0.3. Parameters value: *b*_1_ = 1.0, *b*_2_ = 1.8; *α*_*ij*_ = 1.0; *m*_*i*0_ = 0.1. Initial density *x*_*i*_ = 0.5. See Fig. 1 for software.

**Figure 4:**
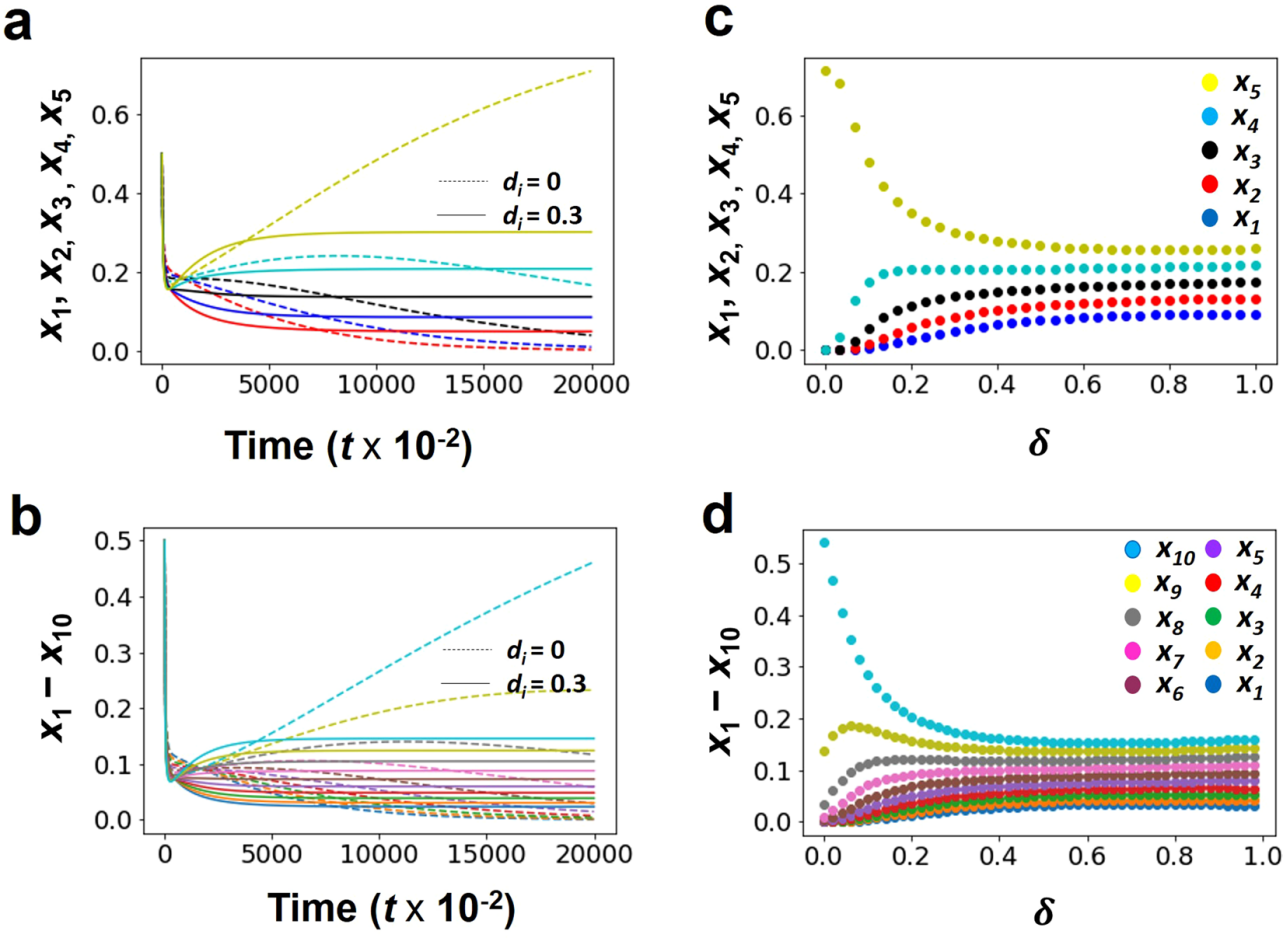
Long-term dynamics of many-species LV competition model with crowding effect. (**a**,**b**) Temporal dynamics with the basic crowding component constant (solid line: *d*_*i*_ = 0.3; dotted line (no crowding): *d*_*i*_ = 0.0) and the crowding strength factor *δ* = 0.3, (**c**,**d**) the equilibrium (final) states plotted against the effects of crowding strength factor *δ* (0 ≤ *δ* ≤ 1). (**a**,**c**) 5 species (birth rate: *b*_*i*_ = 1.0 + 0.1(*i* − 1)); (**b**,**d**) 10 species (birth rate: *b*_*i*_ = 1.0 + 0.05(*i* − 1)). Density of *i*-species: *x*_*i*_ (*i* = 1, 2, …, *n*). Parameter value: *α*_*ij*_ = 1.0; *m*_*i*0_ = 0.1. Initial density *x*_*i*_ = 0.5. See Fig. 1 for software.

Curved lines in the ${x}_{1}-{x}_{2}$ plane imply that coexistence only take place when the value of *δ* → 1.0 for all *d*_*i*_ > 0 (Fig. 2). However, by increasing *δ*, the competitive exclusion reappears in all exclusion cases, where the equilibrium state in ${x}_{1}-{x}_{2}$ plane is strongly curved to return to the originated axis (Fig. 2 except 2c). In contrast, an isocline is straight in ${x}_{1}-{x}_{2}$ plane, where the two species coexist without crowding effects, such that ${K}_{2} < \frac{{K}_{1}}{{\alpha }_{12}}$ and ${K}_{2} < \frac{{K}_{2}}{{\alpha }_{12}}$ (Fig. 2c). Furthermore, Fig. 3a shows that two competing species can coexist for any small positive value of *δ* for all *d*_*i*_> 0. Moreover, small positive real number *δ* will make coexistence possible but bigger value indicates the competitive exclusion principle again (Fig. 2 except 2c and Fig. 3). Figure 4 shows many-species Lotka-Volterra competition dynamics with crowding effects. Temporal dynamics of all species becomes convergent stable by adding crowding effects (Fig. 4a,b). The effect of *δ* in 5 or 10 species (Fig. 4c,d) is qualitatively same with the case of two species competition (Fig. 3b). Thus, many-species LV competition model with crowding effects leads to the stable coexistence of all species (Fig. 4).

**Figure 5:**
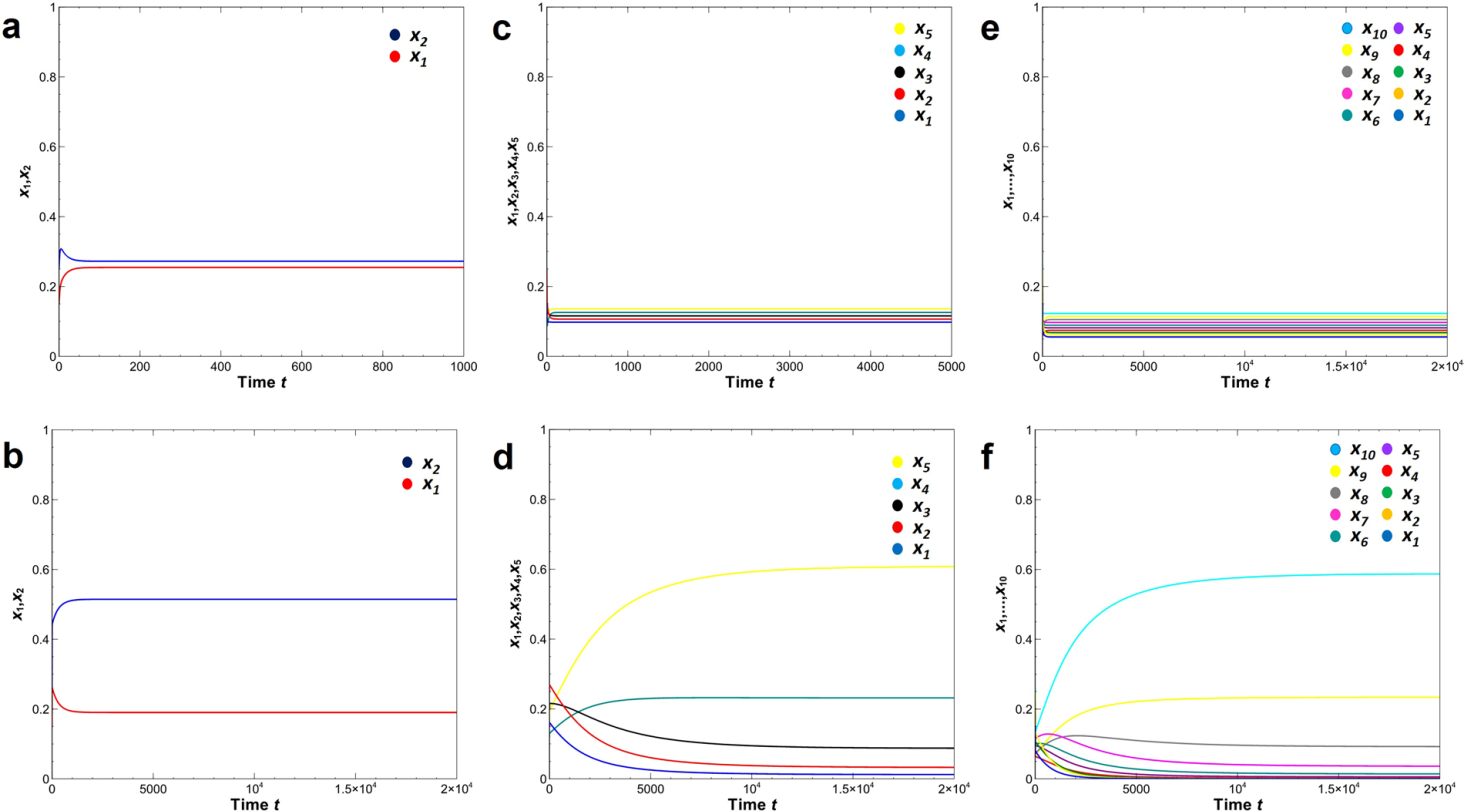
Long-term dynamics of 2-, 5- and 10-species LV competition models with two types of nonlinear density effects. (**a**,**c**,**e**) Crowding effect (Eq. 1; *d*_*i*_ = 0.3, *δ* = 0.4); (**b**,**d**,**f**) aggregation effect (Eq. 3; ε = 0.01). (**a**,**b**) 2 species (*m*_*i*0_ = 0.3); (**c,d**) 5 species (*m*_*i*0_ = 0.3); (**e,f**) 10 species (*m*_*i*0_ = 0.1). Parameter value: *α*_*ij*_ = 1.0; *b*_*i*_ = 1.0 + 0.01(*i* − 1). Initial density: (**a**,**b**) *x*_1_ = 0.15, *x*_2_ = 0.25, (**c**,**d**) *x*_3_ = 0.2, *x*_4_ = 0.12, *x*_5_ = 0.18, (**e**,**f**) *x*_6_ = 0.19, *x*_7_ = 0.22, *x*_8_ = 0.14, *x*_9_ = 0.13, *x*_10_ = 0.26. See Fig. 1 for software.

We also build LV competition models with aggregation effects on mortality that are qualitatively equivalent to the aggregation model of Hartley and Shorrocks^8^. We compare them with the current crowding models using the same parameter conditions (Fig. 5). In the 2-and 5-species dynamics, all species survive and converge to a stable equilibrium in both the crowding model (Fig. 5a,c) and aggregation model (Fig. 5b,d). In the 10-species dynamics, all species survive and converge to stable equilibrium in the crowding models (Fig. 5e), while only five species in the aggregation model.

**Figure 6:**
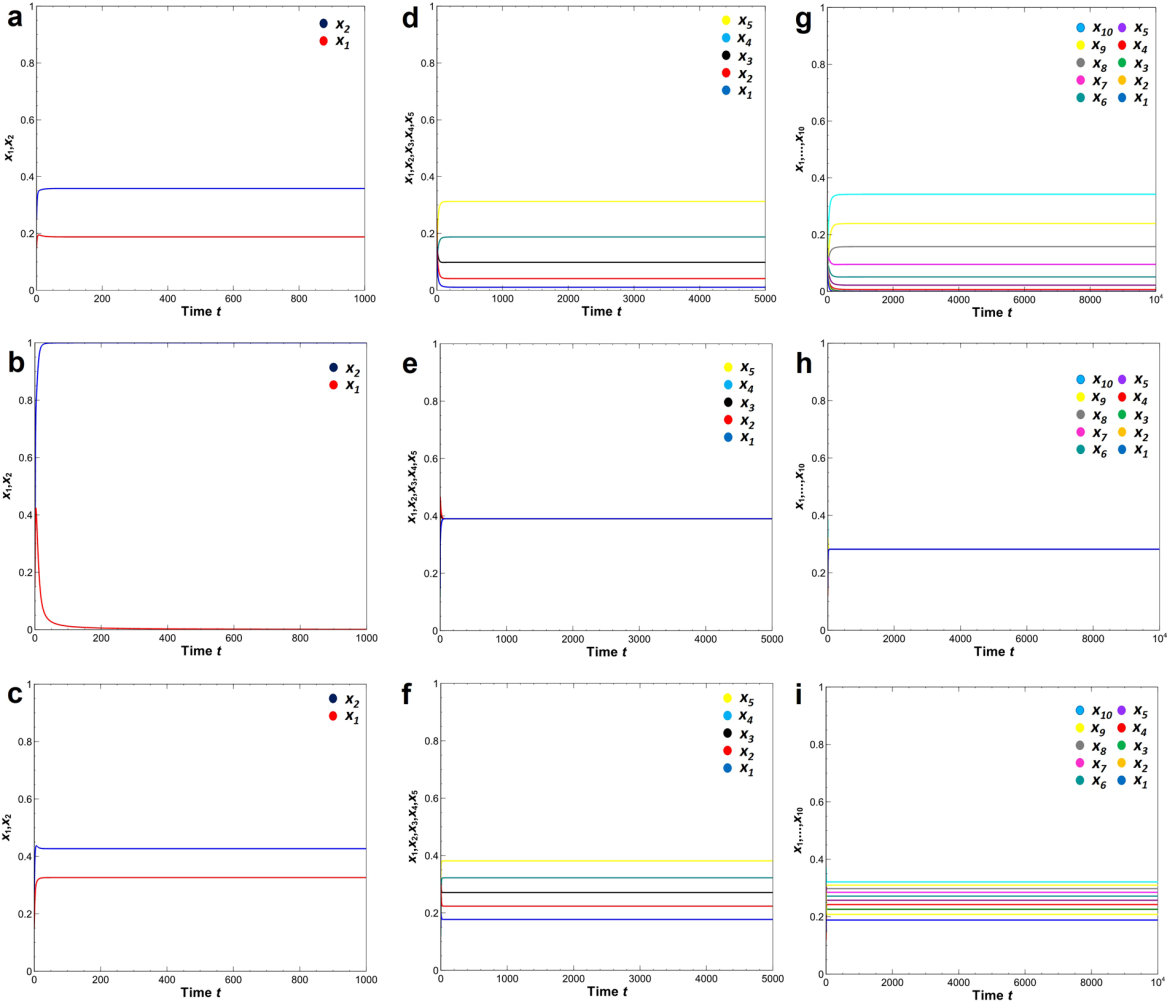
Long-term dynamics of 2-, 5- and 10-species LV competition models with crowding effect and/or nonlinear competition term. (**a**,**d**,**g**) Crowding effect (Eq. 1); (**b**,**e**,**h**) nonlinear competition term; (**c**,**f**,**i**) combination of both (Eq. 3). Parameter value: *α*_*ij*_ = 1.0; *b*_*i*_ = 1.0 + 0.1(*i* − 1). (**a,c,d,f,g,i**) *d*_*i*_ = 0.3, *δ* = 0.4. (**a–f**) *m*_*i*0_ = 0.3; (**g–i**) *m*_*i*0_ = 0.1. Initial density: (**a–c**) *x*_1_ = 0.15, *x*_2_ = 0.25, (**d–f**) *x*_3_ = 0.2, *x*_4_ = 0.12, *x*_5_ = 0.18, (**g–i**) *x*_6_ = 0.19, *x*_7_ = 0.22, *x*_8_ = 0.14, *x*_9_ = 0.13, *x*_10_ = 0.26. All simulations run for the same time steps. See Fig. 1 for software.

We also consider the effect of nonlinear competition terms^21^ with or without crowding effect. We compare them with the current crowding models using the same parameter conditions (Fig. 6). In their original model, Taylor and Crizer consider a modified Lotka-Volterra model introducing the effect of nonlinear competition on growth rate. Here, we introduce the nonlinear competition on birth rate alone, since crowding effect is introduced only on mortality rate. In this manner, we can compare the effect of these changes separately. In the 2-species dynamics, both species survive and converge to a stable equilibrium in the crowding model with linear or nonlinear competition terms (Fig. 6a,c). In the current parameter conditions, the LV competition model with nonlinear competition terms lead to the extinction of one species (Fig. 6b). Note that by changing the conditions, this model lead to the coexistence of the two species^21^. In the 5-species dynamics, all five species survive and converge to a stable equilibrium (Fig. 6d,e and f). Interestingly, the model with nonlinear competition terms converges to the same density for all five species (Fig. 6e), while the crowding effect lead to different densities among all species (Fig. 6d,f). In the 10-species dynamics, seven species survive and converge to stable equilibrium in the crowding models (Fig. 6g). In contrast, all ten species in the LV competition model with nonlinear terms lead to the coexistence of all species with an equal density (Fig. 6h). By combining the nonlinear competition and the crowding effect, all ten species survive and converge to different densities (Fig. 6i).

## Discussion

Many species compete for precisely the same limited resources to survive^1,2^. Gause’s exclusion principle show that multiple competing species cannot coexist in natural communities^1,4,18,19^. Only one species, the superior competitor, will survive and other competitors will eventually become extinct. We should note that frequency dependence does not promote the coexistence of multiple species^20^. Niche theory suggested that it will only become possible for the competing species to coexist if they have different niche^2,3^. Linear density effects show that coexistence becomes possible under very limited conditions. Hence, we search for mechanisms that will enable coexistence of competitive species^5,6^. As a universal and more biologically founded solution, we consider crowding effect, nonlinear density effects at high densities, in the LV competition systems.

Classical LV competition model shows that it is only possible for two species to coexist together if intraspecific competition is stronger than interspecific competition^3^. However, our results have shown that inclusion of crowding effect to the classical Lotka-Volterra competition system guarantees the coexistence of two or more species. We have investigated that two species can coexist when we include crowding effect to the classical LV competition system (Fig. 1 (solid lines), Fig. 2 as *δ* → 1.0, Fig. 3a as *δ* → 1.0, and Fig. 4a,b (solid lines)) compare to the results of the classical LV competition model which show the competitive exclusion principle (Fig. 1 (dotted lines) and Fig. 4a,b (dotted lines)).

Crowding effect has been recognized in many natural and experimental populations^7–16^. Note that if the density of population is increased a lot more than the carrying capacity, then crowding effect will kill all competing individuals. We here, introduced the intraspecific crowding effect into the Lotka-Volterra competition model. We have shown that a weak crowding effect make it possible to achieve a stable coexistence of multiple species. Our analysis implies that increased mortality under high density works as elevated intraspecific competition leading to the coexistence. This may be another ubiquitous mechanism for the coexistence of multiple species leading species diversity in nature.

We compare the current model with the aggregation model of coexistence^8^ (Fig. 5). Unlike the original aggregation model of Hartley and Shorrocks in which the aggregation reduces growth rates, we only include the aggregation effect on mortality rate. These examples show that the aggregation model becomes difficult to maintain the coexistence of all or many species when the number of species is increased. In contrast, the coexistence of all or many species can be easily achieved in the current crowding model even when the number of species is increased (Fig. 5a,c and e). The reason why the coexistence becomes difficult when the number of species increased in the aggregation model seems to depend on the combination of the parameters, where the coexistence region is expressed as a polygon in the *n*-species parameter space which can disappear easily when the number of species is increased. The logic is same with linear programming in *n*-dimensional space. In contrast, in the crowding model, the mortality rate of any species increases when its density approaches its carrying capacity. Because of this, increasing mortality rate near carrying capacity will keep all the species at the densities much below their carrying capacity. Thus, the intraspecific competition becomes most severe at or near carrying capacity, resulting in the stable coexistence of all species.

We also compare the effects of the nonlinear competition terms with the current crowding effects (Fig. 6). Unlike the LV model with nonlinear competition effects on growth rates^21^, we only include the nonlinear competition effects on birth rate alone since crowding effect is included only on mortality rate, so that these effects can be easily distinguished. In the current parameter conditions, the 2-species model results in the exclusion of one species (Fig. 6b). However, when the number of species is increased, LV model with nonlinear competition terms will lead to the coexistence of all species with an equal density regardless of the parameter combinations (Fig. 6e,h). It is not sure whether the stability can be achieved easily when the number of species increased in the nonlinear competition model. However, the equilibrium density is identical for all coexisting species in the model with nonlinear competition terms. This means that the effects of other species-specific parameters are completely cancelled by the introduction of nonlinear competition effect. In contrast, the coexistence of all or many species can be easily achieved in the current crowding model even when the number of species is increased (Fig. 6a,d and g). By combining the nonlinear competition and the crowding effect, many species survive and converge to different densities (Fig. 6c,f and i). Thus, both mechanisms can promote the coexistence of many species differently.

The most important assumption in our model of crowding effect is the increase in mortality rate at high density. Here, *d*_*i*_ represents the proportion of crowding mortality contribution and *δ* is the power of crowding effect. Therefore, when *d*_*i*_ = 0, this model reduces to the classical LV competition model. This assumption should be one natural way to include the crowding effect. However, there may be many other natural ways to include crowding effect, e.g., the aggregation model of Hartley and Shorrocks^8^. We have to wait for empirical studies to verify which way is actually functioned in a natural ecosystem. Note that these functional mechanisms are not exclusive of each other. Thus, the valid mechanisms may be different depending on a natural ecosystem. We should also note that the nonlinearity in the functional responses should be an important factor driving population dynamics and resulting evolution. The nonlinearity in density effect may be biologically inherent and appears as crowding effect at high densities and as Allee effect at low densities. Our studies thus show that the real competitive communities have a much more complicated dynamical system than the classical LV competition system.

## Methods

### Mathematical models

We consider the modified Lotka-Volterra (LV) competition equations with crowding effect rate ***m***_***i***_ for species *i*. In our model, we only consider competition between two species. In addition, carrying capacity ***K***_***i***_ is set to be equal to 1, i.e., *K*_*i*_ = 1. The modified LV competition model is shown on the following equations:

1 $$\{\begin{array}{c}\frac{d{x}_{1}}{dt}={b}_{1}{x}_{1}(1-{x}_{1}-{\alpha }_{12}{x}_{2})-{m}_{1}{x}_{1},\quad {b}_{1}\,{\rm{and}}\,{\alpha }_{12}\,{\rm{are}}\,{\rm{constant}}\\ \frac{d{x}_{2}}{dt}={b}_{2}{x}_{2}(1-{x}_{2}-{\alpha }_{21}{x}_{1})-{m}_{2}{x}_{2},\quad {b}_{2}\,{\rm{and}}\,{\alpha }_{21}\,{\rm{are}}\,{\rm{constant}}\end{array}$$

where ***x***_***i***_ represents the population density of species *i* where *i* = 1, 2. In this model, parameter ***b***_***i***_ represents the birth rate of species *i* while ***α***_***ij***_ represents the effect of species *j* on *i* where *i*,*j* = 1, 2 and *i* ≠ *j*. The crowding effect rate *m*_*i*_ is given by

2 $${m}_{i}={m}_{i0}+{d}_{i}{x}_{i}^{\delta },\quad \delta \in (0,\infty )\forall i$$

where parameter ***m***_***i***0_ represents the initial mortality factor of species *i*. Parameter ***d***_***i***_ represents the density-dependent factor of species *i*. In addition, the sum of the initial mortality and density-dependent factor of species *i* must be greater than 0 but less than or equal to 1, i.e.,0 < *m*_*i*0_ + *d*_*i*_ ≤ 1. Note that, if the initial mortality factor *m*_*i*0_ is zero then nonlinear crowding effect rate *m*_*i*_ will imply that the intraspecific competition is perfectly density-dependent. In addition, if *d*_*i*_ = 0 ${\rm{\forall }}i$ then *m*_*i*_ = *m*_*i*_0 ${\rm{\forall }}i$  which will imply that the modified LV competition model is the same with the classical LV competition model.

Following equations 4 and 11 in the paper of Hartley and Shorrocks^8^, we arrived with the Lotka-Volterra competition model adding the effect of a few more individuals, shown on the following equations:

3 $$\{\begin{array}{c}\frac{d{x}_{1}}{dt}={b}_{1}{x}_{1}(1-{x}_{1}-{\alpha }_{12}{x}_{2})-{m}_{10}{{x}_{1}}^{1+\varepsilon }\,,\quad {b}_{1}\,{\rm{and}}\,{\alpha }_{12}\,{\rm{are}}\,{\rm{constant}}\\ \frac{d{x}_{2}}{dt}={b}_{2}{x}_{2}(1-{x}_{2}-{\alpha }_{21}{x}_{1})-{m}_{20}{{x}_{2}}^{1+\varepsilon },\quad {b}_{2}\,{\rm{and}}\,{\alpha }_{21}\,{\rm{are}}\,{\rm{constant}}\end{array}$$

where *ε* is any positive real number and ${m}_{i0}{{x}_{i}}^{1+\varepsilon }$ is the effect of a few more individuals for all species *i*. Note that, we do not include the crowding effect on birth rates unlike the aggregation model of Hartley and Shorrocks^8^.

In addition, we also used the modified LV competition model of Taylor and Crizer^21^ with the inclusion of nonlinear crowding effect for two species. In their model, they add nonlinear competition terms to prevent the population of species 2 to have a smaller effect on the population of species 1 when the population density of species 1 is very small compare to the population density of species 2 and vice versa. Taylor and Crizer’s competition model with nonlinear crowding effect is shown on the following equations:

4 $$\{\begin{array}{c}\frac{d{x}_{1}}{dt}={b}_{1}{x}_{1}(1-{x}_{1}-{\alpha }_{12}{{x}_{2}}^{2})-({m}_{10}+{d}_{1}{{x}_{1}}^{\delta }){x}_{1}\,,\quad {b}_{1}\,{\rm{and}}\,{\alpha }_{12}\,{\rm{are}}\,{\rm{constant}}\\ \frac{d{x}_{2}}{dt}={b}_{2}{x}_{2}(1-{x}_{2}-{\alpha }_{21}{{x}_{1}}^{2})-({m}_{20}+{d}_{2}{{x}_{2}}^{\delta }){x}_{2},\quad {b}_{2}\,{\rm{and}}\,{\alpha }_{21}\,{\rm{are}}\,{\rm{constant}}\end{array}.$$

### Numerical simulations

In order to determine the impact of the inclusion of nonlinear crowding effects to the classical Lotka-Volterra equation we simulate the modified LV competition equations using Anaconda package of the software Python 3.6^22^. Initially, we determine its effect if there are two competing species in a community and later extend it up to 10 competing species. We also determine the effect when we use small and large values of δ. Moreover, we identify the right combination of *d*_*i*_ and δ that will allow competing species to coexist. Without losing essential qualitative dynamics, we considered the following parameter ranges in our numerical simulations:

- 0 ≤ Initial of *x*_*i*_ ≤ 1 for all *i*;
- 0 < *α*_*ij*_ ≤ 1 for all *i*, *j*;
- 1 ≤ *b*_*i*_ ≤ 2 for all *i*
- *m*_*i*0_ = 0.1 or 0.3 for all *i*;
- *d*_*i*_ = 0,0.1,0.3 or 0.5 for all *i*;
- 0 ≤ δ ≤ 5; and
- *K*_*i*_ = 1

In addition, we compare the results of the LV competition equation (1) with nonlinear crowding effect (${m}_{i}={m}_{i0}+{d}_{i}{x}_{i}^{\delta }$) and without nonlinear crowding effect (*m*_*i*_ = *m*_*i*0_) using the four possible cases of isoclines. The four isocline cases are:

- $${K}_{1} < \frac{{K}_{2}}{{\alpha }_{21}},{K}_{2} < \frac{{K}_{1}}{{\alpha }_{12}}$$
- $${K}_{1} < \frac{{K}_{2}}{{\alpha }_{21}},{K}_{2} > \frac{{K}_{1}}{{\alpha }_{12}}$$
- $${K}_{1} > \frac{{K}_{2}}{{\alpha }_{21}},{K}_{2} < \frac{{K}_{1}}{{\alpha }_{12}}$$
- $${K}_{1} > \frac{{K}_{2}}{{\alpha }_{21}},{K}_{2} > \frac{{K}_{1}}{{\alpha }_{12}}$$

where *K*_*i*_ is the carrying capacity of species *i* and *α*_*ij*_ represents the effect of species *j* on *i* where *i*, *j* = 1, 2 and *i* ≠ *j*.
